# miRNA-940 reduction contributes to human Tetralogy of Fallot development

**DOI:** 10.1111/jcmm.12309

**Published:** 2014-06-01

**Authors:** Dandan Liang, Xinran Xu, Fangfei Deng, Jing Feng, Hong Zhang, Ying Liu, Yangyang Zhang, Lei Pan, Yi Liu, Dasheng Zhang, Jun Li, Xingqun Liang, Yunfu Sun, Junjie Xiao, Yi-Han Chen

**Affiliations:** a Key Laboratory of Basic Research in Cardiology of the Ministry of Education of China, East Hospital, Tongji University School of MedicineShanghai, China; b Institute of Medical Genetics, Tongji UniversityShanghai, China; c Department of Cardiology, East Hospital, Tongji UniversityShanghai, China; d Cardiothoracic Surgical Department, The First Affiliated Hospital of Nanjing Medical UniversityNanjing, China; e Department of Pathology and Pathophysiology, Tongji University School of MedicineShanghai, China

**Keywords:** Tetralogy of Fallot, microRNA, human cardiomyocyte progenitor cell

## Abstract

Tetralogy of Fallot (TOF) is a complex congenital heart defect and the microRNAs regulation in TOF development is largely unknown. Herein, we explored the role of miRNAs in TOF. Among 75 dysregulated miRNAs identified from human heart tissues, miRNA-940 was the most down-regulated one. Interestingly, miRNA-940 was most highly expressed in normal human right ventricular out-flow tract comparing to other heart chambers. As TOF is caused by altered proliferation, migration and/or differentiation of the progenitor cells of the secondary heart field, we isolated Sca-1^+^ human cardiomyocyte progenitor cells (hCMPC) for miRNA-940 function analysis. miRNA-940 reduction significantly promoted hCMPCs proliferation and inhibited hCMPCs migration. We found that *JARID2* is an endogenous target regulated by miRNA-940. Functional analyses showed that *JARID2* also affected hCMPCs proliferation and migration. Thus, decreased miRNA-940 affects the proliferation and migration of the progenitor cells of the secondary heart field by targeting *JARID2* and potentially leads to TOF development.

## Introduction

Congenital heart defect is the most common birth defect in humans with a prevalence of ∼1% of all live births [1,2]. Tetralogy of Fallot (TOF), consisting of pulmonary stenosis, ventricular septal defect, overriding aorta and right ventricular hypertrophy, is the most common cyanotic congenital heart defect and accounts for about 10% of all congenital heart malformations [3,4]. Besides occurring as an isolated defect, TOF has also been associated with deletions on chromosome 22 and with diGeorge syndrome [1,2]. The development and refinements of surgical repair techniques for TOF have led to a steadily increasing number of adult survivors. Despite these advances, the underlying molecular mechanisms of TOF remain elusive [1,3].

Organ patterning during embryonic development requires precise temporal and spatial regulation of gene expression and protein activity [5,6]. MicroRNAs (miRNAs) are a novel class of endogenous 22-nucleotide-long non-coding RNAs that regulate gene expression at the post-transcriptional level [7] in many essential biological process including cell growth, proliferation, differentiation, apoptosis and development [8–12]. Regulation by miRNAs has been shown to be broadly important for proper embryonic development [5,6,13]. For example, studies showed that knock-out miRNA-1-2 in mice resulted in fatal septal abnormalities together with thickened ventricular walls caused by persistent proliferation [14]. Overexpression of miRNA-1 in Drosophila led to embryonic lethality because of an insufficient number of cardioblasts [15]. Similar to miRNA-1, the co-expressed miRNA-133 has also been shown to be necessary for atrioventricular canal development in zebrafish [13]. In addition, deletion of the miRNA-17–92 cluster has been reported to produce ventricular septal defects in mice [16]. Moreover, decreased miRNA-138 led to expansion of atrioventricular canal gene expression into the ventricular chamber and failure of ventricular cardiomyocytes to fully mature in zebrafish [17].

Tetralogy of Fallot is the result of altered proliferation, migration and/or differentiation of the progenitor cells of the secondary heart field [18]. Islet-1 has been proposed as a marker of cardiac progenitor cells derived from the second heart field and is utilized to identify and purify cardiac progenitors from murine and human specimens for *ex vivo* expansion [19]. Islet-1 was shown to be downregulated as these cells differentiate and contribute to the elongating heart tube [19]. Islet-1 is required for the process of heart tube elongation; in Islet-1 mutant embryos the linear heart tube fails to extend and loop, and BMP and FGF gene expression is downregulated in the distal heart tube and pharyngeal region [19].

Tetralogy of Fallot is a manifestation of developmental abnormality, yet the involvement of miRNAs remains unclear. In the present study, we sought to explore the role that miRNAs play in human TOF development.

## Materials and methods

### Human tissue preparation

This study was approved by the ethical committees of the Tongji hospital, Tongji University School of Medicine (Protocol Number: LL (H)-10-02-2). Right ventricular out-flow tract tissues, which were considered to be surgical waste, were obtained during heart surgery from TOF patients. TOF was diagnosed by using recognized morphological criteria (anterior deviation of the infundibular septum with ventricular septal defect and obstruction to right ventricular out-flow tract) and all the samples had typical morphology and were therefore considered to be classic TOF. Normal tissue samples from the right ventricular out-flow tract, specifically the proximal ventricular tissue, were obtained from healthy, prospective multi-organ donors without cardiovascular pathology who could not be transplanted because of technical reasons. For the screen experiment, 10 TOF patients (adults) and eight healthy adult control samples were used. For the validation experiments, an independent set of samples including 26 TOF patients and 15 healthy individuals were tested.

### miRNA arrays analysis

Total RNA was isolated from human right ventricular out-flow tracts by using the mirVana™ miRNA Isolation kit (Ambion Inc., Austin, TX, USA), according to the manufacturer's instructions. The RNA quality for each sample was checked by using an Agilent 2100 Bioanalyzer (Agilent Technologies, Santa Clara, CA, USA). 100 ng total RNA was dephosphorylated and ligated with pCp-Cy3. Labelled RNA was purified and hybridized to Agilent human miRNA arrays (V2), which cover 723 human and 76 human viral miRNAs from the Sanger database v.10.1 (Agilent Technologies, Foster City, CA, USA) [11]. Hybridization was carried out at 55°C for 20 hrs; the arrays were washed and scanned on Agilent Scan Control software and were then analysed with the Agilent Feature Extract software version 9.5.3. The expression threshold was set at the average signal intensity detected in samples without input miRNA. miRNA expression data were normalized by bead-based assay by using the locally weighted smooth spline (LOWESS) method. After normalization, all expression values were transformed to a linear scale for statistical comparisons.

### Quantification of miRNA expression

To validate that miRNA-940 is cardiac specific or enriched, total RNAs from human tissues including lung, pancrease, heart, brain, kidney, skeletal muscle, spleen, liver, bladder, stomach, colon, intestine (Applied Biosystems) were separated on a 10% acrylamide TBE-urea mini-gel (Applied Biosystems) and then electroblotted onto Hybond N+ nylon filter (Amersham Biosciences, Amersham, UK). An end-labelled (Exiqon, Vedbaek, Denmark) oligonucleotide probe for miRNA-940 (GGGGAGCGGGGGCCCTGCCTT) was hybridized to the filter in Rapidhyb buffer (Amersham Biosciences). The blot was reprobed for U6 to control for equal loading according to the manufacturer's protocol (Amersham Biosciences).

### Human cardiomyocyte progenitor cells isolation and differentiation

Human cardiomyocyte progenitor cells (hCMPCs) were isolated as previously described [12,20,21]. In brief, human foetal heart tissue from elective abortion was collected and cut into small pieces after, followed by collagenase and protease treatment. A single cell suspension was obtained by passing through a cell strainer and hCMPCs were isolated *via* flow cytometry by using a mouse anti-stem cell antigen-1(Sca-1) antibody (eBioscience, San Diego, CA, USA) and characterized as previously described [20]. hCMPCs were cultured and passaged as described elsewhere [20]. Islet-1 was stained by using a polyclonal rabbit anti-Islet-1 (Abcam, Cambridge, England) and a second antibody Alexa Fluor 555 goat anti-rabbit (Invitrogen, Carlsbad, CA, USA). Images were taken by using Live CELL Imaging System (Leica AF 7000; Leica, Solms, Germany). To induce cardiomyocytes differentiation, hCMPCs were treated with 5-azacytidine (Sigma-Aldrich, St. Louis, MO, USA) treatment (5 μM) and ascorbic acid (Sigma-Aldrich; 100 μM) for up to 14 days. To induce smooth muscle cells differentiation, hCMPCs were treated with TGF-beta1 (PeproTech, Rocky Hill, CT, USA; 1 ng/ml) for 6 days.

### miRNA-940 transfection experiments

miRNA-940 function was explored by cell transfection experiment. miRNA-940 mimics (Cat. no. MSY0004983), inhibitor (Cat. no. MIN0004983) and negative control (Cat. no. MSY0002505) were purchased from Qiagen(Venlo, Limburg, the Netherlands). hCMPCs were transfected with siPORT™ NeoFX™ Transfection Agent (Ambion) and miRNA-940 mimics, inhibitor and negative control, according to the manufacturer. A serial of transfection concentrations was tested and 30 nM was chosen for the study. The transfection efficiency was confirmed by RT-PCR and visually by means of FAM-labelled negative control. For fluorescence microscope analysis of FAM-labelled pre-miR expression, cells were washed with PBS and fixated with 4% paraformaldehyde in PBS for 15 min. at RT. Nuclei were stained with 0.2 μg/ml DAPI (Invitrogen).

### hCMPCs proliferation, viability and apoptosis/necrosis

Cells were transfected with 30 nM miRNA-940 mimics, miRNA-940 inhibitor or negative control in culture medium containing 1% FBS. After 48 hrs, EdU was added to the culture medium and re-incubated for 24 hrs. Cell proliferation was determined by using the Click-iT Edu Imaging Kits (Invitrogen). Briefly, after removing the culture medium, the cells were fixed with 3.7% formaldehyde and permeabilized with 0.5% Triton-X-100. Click-iT reaction cocktail was added for incubating for 30 min. at room temperature. After washing, DNA was stained with Hoechst 33342 and the images were taken by using Live CELL Imaging System (Leica AF 7000; Leica). A cell proliferation ELISA, BrdU (Roche, Basel, Switzerland) was also used according to the manufacturer as a colorimetric immunoassay for the quantification of cell proliferation, based on the measurement of BrdU incorporation during DNA synthesis.

Cell viability was assessed by using the Cell Counting Kit-8 (CCK-8; Dojin, Tokyo, Japan). Briefly, 10 μl of the CCK-8 solution was added to each well of the plate. The plate was incubated at 37°C for 2 hrs. The absorbance was measured at 450 nm by using the VICTOR™ X5 Multilabel Plate Reader (PerkinElmer, Waltham, MA, USA) with a reference wavelength of 650 nm.

Apoptosis and necrosis were detected by staining for Annexin V and PI (Roche) and followed by MoFlo XDP flow cytometry (Beckman Coulter, Pasadena, CA, USA) analysis as previously described [11].

### hCMPCs differentiation determination

Human cardiomyocyte progenitor cells were transfected with miRNA-940 mimics (30 nM), miRNA-940 inhibitor (30 nM) or the negative control. hCMPCs differentiation were induced according to the procedure as described above. 5-azacytidine (Sigma-Aldrich) was added for 72 hrs in differentiation medium, then hCMPCs were treated with ascorbic acid (Sigma-Aldrich) for 14 days. Two methods were used to confirm the differentiation of hCMPCs into cardiomyocytes as previously described [12,20]. Briefly, qRT-PCRs were performed to detect the expression of cardiac genes including: MEF2C, GATA-4, Nkx-2.5, TropT, bMHC and cActin. Stainings for α-actin by using a monoclonal mouse anti-actinin (sarcomeric) clone EA-53 (Sigma-Aldrich) as well as Nkx2.5 (antibody from Abcam) were performed to quantify the degree of differentiation. To confirm the differentiation of hCMPCs into smooth muscle cells, two independent techniques were used. qRT-PCRs and western blot were used to detect the expression of smooth muscle α-actin (α-SMA) (antibody from Abcam) and calponin (antibody from Santa Cruz Biotechnology, Dallas, TX, USA), given α-SMA is an early marker for smooth muscle cells and calponin is a specific marker for smooth muscle cells.

### hCMPCs migration measurement

Cell migration was determined by using the RadiusTM 24-well cell migration assay kit (Cell Biolabs, Inc., San Diego, CA, USA). Briefly, 500 μl of the hCMPCs suspension at a dosage of 0.3 × 10^6^ cells/ml was transfected with miRNA-940 mimics (30 nM), miRNA-940 inhibitor (30 nM) or the negative control. The RadiusTM Gel was removed after 24 hrs by using 0.5 ml of 1*RadiusTM Gel Removal Solution. The cells were fixed and stained with DAPI after 24 hrs and subsequently examined under microscope. The migration capacity of hCMPCs with miR-940 transfection were further tested by using an *in vitro* migration assay by performing BD (8-μm-pore) transwell assay according to the user's manual. For quantification, the cells were counted by photographing the membrane under a microscope in 10 predetermined fields at 400× magnification.

### miRNA-940 target gene analysis

GOmir (http://www.bioacademy.gr/bioinformatics/projects/GOmir/) was used to identify potential human miRNA-940 target genes. Since TOF was a manifestation of abnormal heart development, we focus those predicted genes that have been shown to affect heart development. *Jumonji, AT rich interactive domain 2* (*JARID2*) was predicted to be a potential target gene for miRNA-940 and it has been shown to regulate out-flow tract morphogenesis. So we focus on *JARID2* for the downstream analysis. Two independent strategies were used to confirm these predicted genes as the targets of miRNA-940. First, a luciferase reporter assay was used to confirm the target gene. Second, hCMPCs transfection experiments were performed followed by western blot to confirm that miRNA-940 could endogenously regulate *JARID2* expression. Experiential details were described in Data S1.

### Modulation of *JARID2* in hCMPCs

*JARID2* sequence was cloned into a pcDNA3 vector for overexpression experiments. Target-specific siRNA oligo (5′-GAGGGCUGAAGUUGAUGUATT UACAUCAACUUCAGCCCUCTT-3′) of human *JARID2* were ordered (GenePharma Co., Shanghai, China) for down-regulation of *JARID2*. hCMPCs were transfected with these constructs and cell proliferation and migration were assessed as described above.

### Statistical analysis

Data are expressed as the mean ± SE. An independent-sample *t*-test, Chi-squared test or one-way anova was conducted to evaluate the one-way layout data whenever appropriate. If a significant difference was found, Bonferroni's post-hoc test was conducted to determine which groups differed significantly. *P*-values <0.05 were considered as statistically significant.

## Results

### miRNA-940 is most significantly down-regulated in TOF patients

**Fig. 1:**
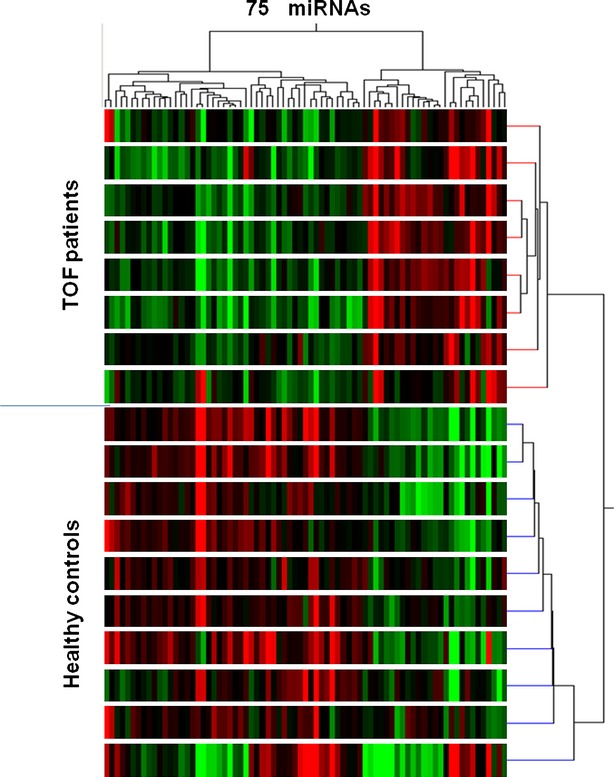
Heat map of the unsupervised hierarchical clustering of the 75 differentially expressed miRNAs. The expression profile of the 75 differentially expressed miRNAs distinguished two groups of samples examined, which correspond to 10 TOF patients and 8 health controls, respectively.

**Fig. 2:**
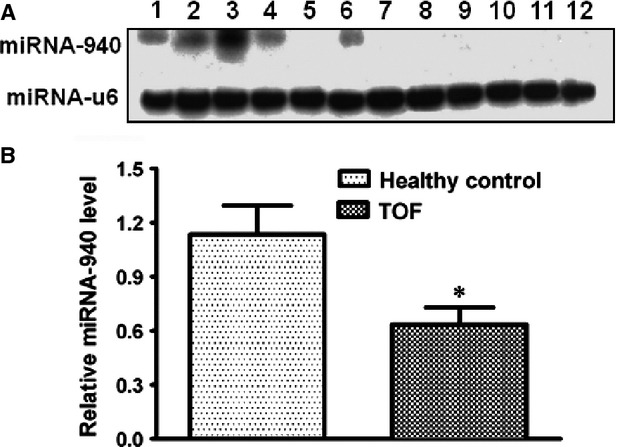
miRNA-940 is the most down-regulated miRNA in TOF patients. (**A**) Northern blot shows that miRNA-940 is highly expressed in human heart. Shown in each lane is: 1-lung; 2-pancrease; 3-heart; 4-brain; 5-kidney; 6-skeletal muscle; 7-spleen; 8-liver; 9-bladder; 10-stomach; 11-colon; 12-intesetin. U6 was used as a loading control. (**B**) qRT-PCRs validate the down-regulation of miRNA-940 in an independent set of 26 TOF patients comparing to 15 healthy controls. *P < 0.05.

**Fig. 3:**
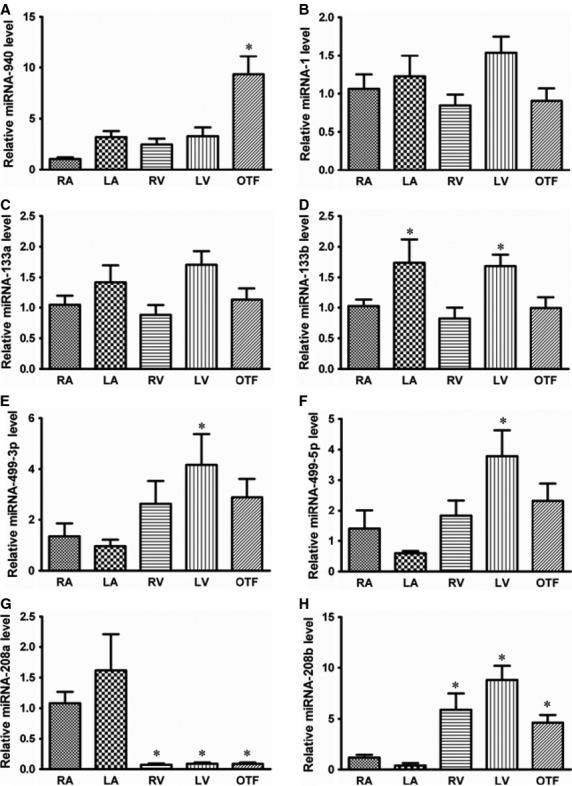
miRNA-940 is most abundant in human right ventricular out-flow tract. Expression profile of (**A**) miRNA-940; (**B**) miR-1; (**C**) miR-133a; (**D**) miR-133b; (**E**) miR-499-3p; (**F**) miR-499-5p; (**G**) miR-208a; (**H**) miR-208b in different part of human hearts. RA, right atria; LA, left atria; RV, right ventricle; LV, left ventricle; OTF, right ventricular out-flow tract. *P < 0.05, compared to RA, *n* = 6.

The human miRNA arrays identified 75 differentially expressed miRNAs among ∼800 examined miRNAs between the 10 TOF patients and eight healthy controls. Fold-changes of these differentially expressed miRNAs were shown in Table S1. 27 out of the 75 miRNAs were down-regulated in TOF patients compared to healthy controls, while 48 miRNAs were up-regulated. miRNA-940 was the most down-regulated one with a more than fourfold change between the two examined groups while miRNA-204 was the most up-regulated one (∼3.6-fold). Unsupervised hierarchic clustering of TOF and control samples was performed for these 75 differentially expressed miRNAs. The heat map of miRNA expression defined two well-defined clusters corresponding to samples from TOF patients and healthy controls (Fig. 1). The tissue expression pattern of human miRNA-940 was shown in Figure 2A. The down-regulation of miRNA-940 in TOF was validated in 26 independent tissue samples from the right ventricular out-flow tract of patients with TOF (Fig. 2B). Furthermore, compared to well-known cardiac or muscle specific miRNAs (miR-1, miR-133a, miR-133b, miR-208a, miR-208b, miR-499-5p and miR-499-3p), miRNA-940 was the only one which is most highly expressed in the normal human right ventricular out-flow tract comparing to other chambers within the heart (Fig. 3). There results indicated that miRNA-940 may be associated with human TOF.

### Decreased miRNA-940 promotes hCMPCs proliferation and inhibits hCMPCs migration

**Fig. 4:**
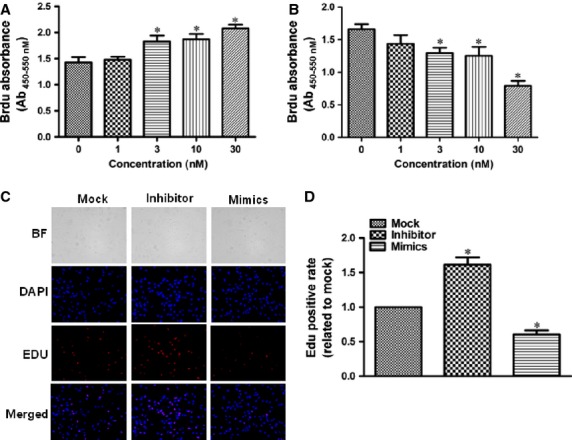
Effects of miRNA-940 mimics and inhibitors on hCMPCs proliferation. Quantification of transfection of miRNA-940 inhibitor (**A**) and miRNA-940 mimics (**B**) in a serial concentrations from seven independent experiments. (**C**) Representative images of miRNA-940 modulation on hCMPCs proliferation as assessed by counting numbers of EdU incorporated cells (×200). (**D**) Quantification of the effects of miRNA-940 mimics and inhibitors on hCMPCs proliferation. *P < 0.05, compared to control, *n* = 8.

As above mentioned, altered proliferation, migration and/or differentiation of the progenitor cells of the secondary heart field lead to TOF development [18]. So we investigated whether miRNA-940 modulation affects these aspects of the hCMPCs. We first confirmed that the isolated Sca-1^+^ hCMPCs express Islet-1, a marker of cardiac progenitor cells derived from the second heart field (Fig. S1). Transfection efficiency was shown in Figure S2. We tested serial concentrations of miRNA-940 inhibitor or mimics at the 0, 1, 3, 10 and 30 nM. miRNA-940 inhibitor transfection (30 nM) significantly increased hCMPCs proliferation while miRNA-940 mimics transfection (30 nM) led to a significant decrease in cellular proliferation compared with control cells (Fig. 4A and B). We further counted the number of EdU incorporated cells, miRNA-940 inhibitor transfection increased hCMPCs proliferation by 61% while miRNA-940 mimics transfection led to a significant up to 40% decrease in cellular proliferation compared with control cells (Fig. 4C and D). This effect was independent of cell viability or cellular apoptosis/necrosis (Fig. S3 and S4). On the other hand, although miRNA-940 expression level decreased during differentiation of hCMPCs to cardiomyocytes (Fig. S5), modulating miRNA-940 level did not affect hCMPCs differentiation, neither to cardiomyocytes nor to smooth muscle cells. As shown in Figure S6, neither miRNA-940 mimics nor inhibitor transfection changed the expression levels of cardiomyocyte-specific genes including *MEF2C*, *GATA-4*, *Nkx-2.5*, *TropT*, *βMHC* and *cActin* after 2 weeks of differentiation. In addition, stainings for α-actin and Nkx2.5 also indicated that miRNA-940 did not affect hCMPCs differentiation into cardiomyocytes (Fig. S7). Moreover, the qRT-PCRs and western blot for α-SMA and calponin consistently showed that miRNA-940 did not affect hCMPCs' differentiation to smooth muscle cells (Fig. S8).

**Fig. 5:**
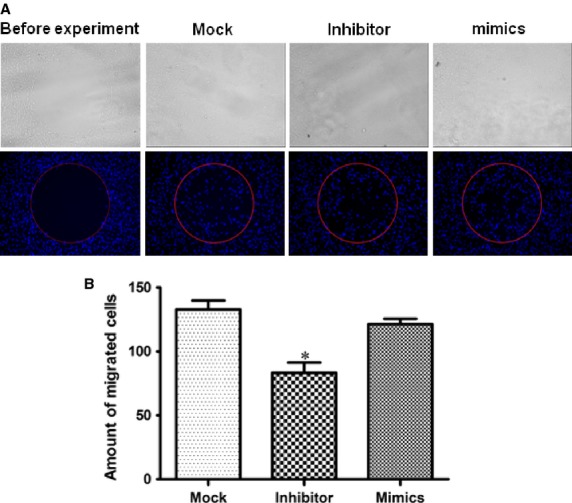
Effects of miRNA-940 mimics and inhibitors on hCMPCs migration. (**A**) Representative images showing the effects of miRNA-940 on hCMPCs migration (×100). (**B**) Quantification of the effects of the miRNA-940 inhibitors on hCMPCs migration. *P < 0.05, compared to mock, n, mock = 7, n, mimics = 6, n, inhibitor = 5.

We examined whether modulating miRNA-940 affect hCMPC migration. As shown in Figure 5 and Figure S9, miRNA-940 inhibitor transfection significantly inhibited migration of hCMPC by 37% while miRNA-940 mimics infection did not affect hCMPCs migration.

### *JARID2* is the target gene of miRNA-940

**Fig. 6:**
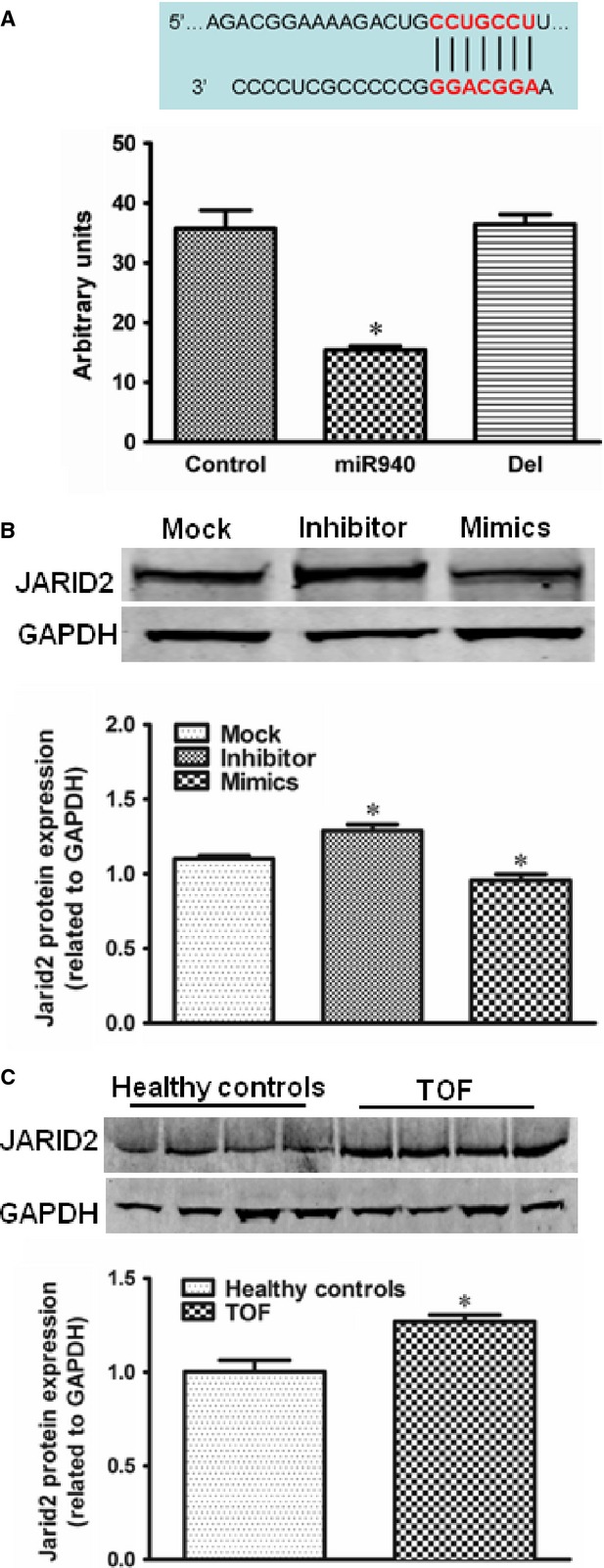
*JARID2* is identified as a target gene of miRNA-940. Quantification of luciferase expression levels are shown for 6 independent experiments for *JARID2* (**A**). *P < 0.05, compared to control. (**B**) Western-blot results and quantification of JARID2 protein level in hCMPCs after negative control, miRNA-940 inhibitor or miRNA-940 mimics transfection. *P < 0.05, compared to control for three independent experiments. (**C**) Western-blot results and quantification of JARID2 protein level in tissues from healthy individuals and TOF patients. Comparisons were between healthy individuals and TOF patients. *P < 0.05, for three independent experiments.

Gomir predicted *JARID2*, a gene having potential effect in cardiac out-flow tract development [22] as a potential target gene for miRNA-940. The luciferase reporter assay indicated that transfection of the luciferase construct containing 3′-UTR of *JARID2* in combination with miRNA-940 mimics led to a significant reduction in luciferase activity (Fig. 6A). The constructs with the miRNA-940 binding site deleted has no effect on the luciferase activity. These results confirm that *JARID2* is a direct target gene of miRNA-940. To confirm that miRNA-940 could endogenously regulate *JARID2* expression, the protein level was evaluated in hCMPCs transfected with miRNA-940 mimics, miRNA-940 inhibitor or negative control. The protein level of *JARID2* was found to be negatively regulated by miRNA-940, indicating that *JARID2* is an endogenous target of miRNA-940 in hCMPCs (Fig. 6B). We further examined the protein levels in tissues from healthy individuals and TOF patients. As shown in Figure 6C, JARID2 protein levels were significantly higher among TOF patients compared to healthy individuals. Such increases again support the negative regulation of miRNA-940 on *JARID2*.

### *JARID2* regulates hCMPCs proliferation and/or migration

**Fig. 7:**
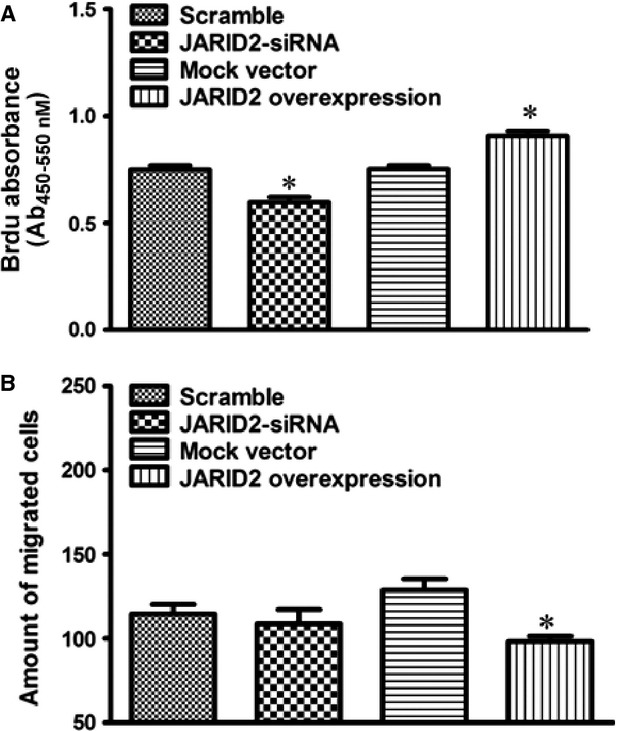
Manipulation of *JARID2* expression in hCMPCs. Measurements were performed after transfection of scramble/interference/mock vectors/overexpression to hCMPCs. (**A**) Quantification of transfection of *JARID2* vectors from seven independent experiments. (**B**) Counting of migrated cells after transfection of *JARID2* vectors from five independent experiments. *P < 0.05, comparisons were made either between overexpression and mock vectors or between interference and scramble vectors.

**Fig. 8:**
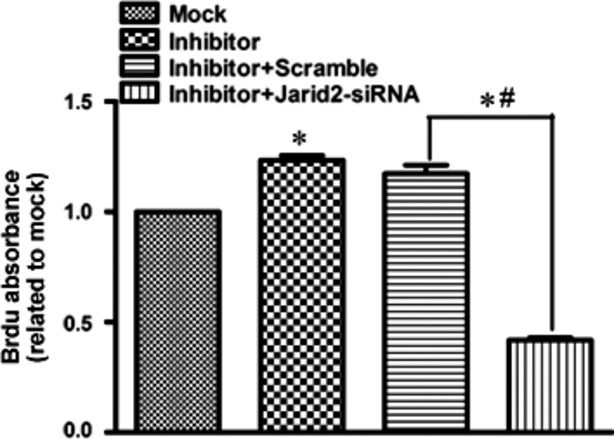
Quantification of the effect on proliferation of *JARID2* siRNA trasnsfection in the miRNA-940 inhibitor transfected hCMPCs. *^#^*P* < 0.05, for five independent experiments.

We manipulated *JARID2* expression in hCMPCs by overexpression and interference constructs transfection. As shown in Figure 7A, overexpression of *JARID2* significantly increased hCMPCs proliferation by 20.4% while *JARID2* interference decreased hCMPCs proliferation by 20.6%. Regarding to the effects on hCMPCs migration, only *JARID2* overexpression significantly inhibited hCMPCs' migration by 23.8% (Fig. 7B). To further clarify JARID2 is a major effector protein among the endogenous target genes of miRNA-940, we down-regulated *JARID2* in the miRNA-940 inhibitor transfected hCMPCs. *JARID2* siRNA trasnsfection cancelled the effect of increase of proliferation of hCMPCs as caused by miRNA-940 inhibitor transfection (Fig. 8).

## Discussion

In this study, we identified a group of differentially expressed miRNAs by microarray screening between TOF patients and healthy individuals, which suggested the involvement of miRNA during the TOF pathogenesis. Among these dysregulated miRNAs, miRNA-940 was the most downregulated one in individuals with TOF. We further determined the spatial expression pattern of miRNA-940. Among three main organs derived from mesoderm, miRNA-940 was most abundant in heart compared to the pancreas and lung. We found that miRNA-940 was highly enriched in human cardiac tissue, particularly in the right ventricular out-flow tract. This expression profile was not observed for a group of well-known cardiac or muscle specific miRNAs (miR-1, miR-133a, miR-133b, miR-208a, miR-208b, miR-499-5p and miR-499-3p). These results indicate an interlink between decreased miRNA-940 and human TOF.

Deletion of the human Chr.22 at the long arm on region 1, band 1, cause several presentations and collectively named 22q11.2 deletion syndrome. Congenital heart disease is one common symptom associated with such deletion and TOF is one of the many presentations. The prevalence of this 22q11.2 deletion among TOF patients varies across ethnicities with an estimated 1:4000 in a Caucasian population [23]. There's no concrete data of such prevalence in Chinese population with few studies reports a less than 15% prevalence [24,25]. We did not have the chr.22 deletion tested for the patients involved in because of some technique difficulties and we would like to acknowledge this as a limitation of our study. Should any patients have deletion of the Chr. 22, the miR-940 function is less likely to be impaired. Plus, given the low prevalence of the Chr.22 deletion among TOF patients, such influence should not be decisive.

Islet-1 has been proposed as a marker of cardiac progenitor cells derived from the second heart field and is utilized to identify and purify cardiac progenitors from murine and human specimens for *ex vivo* expansion. Studies have been shown that cultured Sca-1^+^ cells expressed Islet-1 as well as other early cardiac transcription factors [20,21]. So we isolated Sca-1^+^ hCMPCs as an *in vitro* system to carry out the functional study of the role of miRNA-940 in regulating proliferation, migration and differentiation on the human cardiac derived progenitor cells. We also showed that the isolated Sca-1^+^ expressed Islet-1 and assure us Sca-1^+^ hCMPCs as a proper system to study TOF. It was found that decreased miRNA-940 promoted hCMPCs proliferation, but did not affect cell viability apoptosis or necrosis. Decreased miRNA-940 did not affect differentiation of hCMPCs to either cardiomyocytes or smooth muscle cells as well. Besides promoting proliferation, decreased miRNA-940 also inhibited the migration of hCMPCs. Collectively, these results show that decreased miRNA-940 changed the proliferation and migration of hCMPCs, which potentially contributes to the genesis of TOF.

Identifying the relevant miRNA target genes and building new miRNA-gene networks is the fundamental for the mechanistic study and has always been a challenge [26]. miRNAs act in a complex functional network in which each miRNA might control hundreds of distinct target genes, and the expression of a single coding gene can be regulated by many different miRNAs [7]. We identified that *JARID2* is a target gene of miRNA-940. Study has also shown that Jarid2 is required for normal mice cardiac development by regulate Notch1 signalling *via* histone modification [27] and is among a set of genes differentially regulated by Nkx2.5 during out-flow tract morphogenesis [22]. It regulates gene expression during embryonic development by facilitating the recruitment of the PRC2 complex to target genes [28]. *Jarid2* knock-out mice exhibit cardiac defects including hypertrabeculation with non-compaction of the ventricular wall. Our results showed that *JARID2* could affect hCMPC proliferation and migration and suggested they are regulated by miRNA-940 and involved in cardiac development. However, the precise mechanism of how decreased miRNA-940 contributed to TOF remains unclear. miRNA-940 is only observed in human, macaca mulatta, pan troglodytes and bos taurus, making its extremely hard to generate genetically modified mouse models to study TOF development or progression. Further work is warranted to define the miRNA-940 network.

In our study, adult tissues were used for a screen purpose. miRNA expression levels in the adult may not be the causal factor and tissues from new-born patients may be more relevant. However, the causality of the interested miRNA was further explored in functional study. Our results did provide evidence for the potential pathogenic role of miR-940 in TOF. Compared to other studies reported of miRNAs expression in TOF patients [29,30], the list of the differentially expressed miRNAs reported have some overlap with what we found. The difference could be because of the temporal effects on gene expression as tissues used in our study were from adults while other studied used tissue from young patients or infants.

In summary, we identified that miRNA-940 was significantly down-regulated in TOF patients in comparing with the miRNA expression profiles from human samples. miRNA-940 reduction disturbs the proliferation and migration of the progenitor cells of the secondary heart field, potentially by modulating *JARID2* and ultimately contributing to human TOF. Our finding uncovers an essential role of miRNA-940 in cardiac development and may offer new insight into the pathogenesis of TOF.

## Supporting information

**Data S1** Supplementary materials. **Figure S1** Islet-1 staining of Sca-1^+^ hCMPCs. Isolated Sca-1^+^ hCMPCs was stained for Islet-1 (Isl-1), and DAPI. **Figure S2** Efficiency of the miRNA-940 transfection. **Figure S3** miRNA-940 mimics and inhibitor do not affect human cardiomyocyte progenitor cells viability. **Figure S4** miRNA-940 mimics and inhibitor do not cause apoptosis and necrosis. **Figure S5** miRNA-940 expression during hCMPC differentiation. **Figure S6** miRNA-940 mimics and inhibitor do not affect cardiac-specific gene expression under differentiation condition. **Figure S7** Effects of miRNA-940 mimics and inhibitor on differentiation of hCMPCs to cardiomyocyte. **Figure S8** Effects of miRNA-940 mimics and inhibitors on differentiation of hCMPCs to smooth muscle cells. **Figure S9** Effects of miRNA-940 mimics and inhibitor on hCMPCs migration as assayed by the chamber assay. **Table S1** Differentially expressed miRNAs between Tetralogy of Fallot patients and healthy controls. **Table S2** microRNAs differentially expressed in the heart, pancreas and lung.
