# Supplementary material for: miRNA-940 reduction contributes to human Tetralogy of Fallot development
Source: J Cell Mol Med. 2014 Jun 1;18(9):1830–9. doi: 10.1111/jcmm.12309 (PMC4196658; doi:10.1111/jcmm.12309)
Supplement: Supplementary file 1 [file jcmm0018-1830-SD1.pdf]

Supplementary Materials

Supplemental Methods

Human tissue preparation. Individual permission using standard informed consent procedures was obtained. The tissues were permitted to be used by the Key Laboratory of Basic Research in Cardiology of the Ministry of Education of China, Tongji University School of Medicine. The investigation conforms to the principles outlined in the Declaration of Helsinki regarding the use of human tissues. The tissues were immediately snap-frozen in liquid nitrogen upon collecting and stored at -80°C until further analysis.

miRNA expression analysis. As a preliminary screen and to obtain general trends for miRNA abundance in heart, total RNA of human heart , human pancreas, and human lung (Applied Biosystems, USA), three main organs derived from mesoderm， were analyzed using commercially available miRNA arrays (LC Sciences, Houston, TX) based on miRBase Release 15.

A stem-loop qRT-PCR assay based on SYBR Green I was performed to compare the distribution of miRNA-940 and other well-known cardiac specific/enriched miRNAs including miRNA-1, miRNA-133a, miRNA-133b, miRNA-208a, miRNA-208b, miRNA-499-5p, and miRNA-499-3p in human left atrium (LA), right atrium (RA), left ventricle (LV), right ventricle (RV) and right ventricular outflow tract. The down-regulation of miRNA-940 in TOF was also validated using this qRT-PCR assay in independent tissue samples (right ventricular outflow tract) of

patients with TOF. Following the manufacturer's instruction, cDNA was sythesized using the miScript Reverse Transcription (RT) Kit (Qiagen GmbH, Hilden, Germany). Briefly, 1 μg total RNA, 1 μl miScript Reverse Transcriptase Mix, and 4 μl miScript RT buffer were mixed well and incubated for 60 min at 37 °C. The qRT-PCR was performed on an Mx3000 QPCR System (Stratagene, La Jolla, CA) using the miScript SYBR Green PCR Kit (Qiagen, Valencia, CA). 20 μL PCR reaction system contained 2 μl RT product, 10 μl 2× QuantiTect SYBR Green PCR Master Mix, 2 μl 10× miScript Universal Primer, 2 μl 10× miScript Primer Assay (Qiagen), and 4μl nuclear-free water. The reaction protocol was as follows: 95 °C for 10 min, followed by 40 amplification cycles of 94 °C for 15 s, 55 °C for 30 s, and 70 °C for 30 s. All qRT-PCR reactions, including no-template controls, were performed in triplicate. The relative expression ratios of miRNAs were determined with the crossing point as the cycle number. A highly conserved and universally expressed small nRNA, U6, was used as endogenous controls. The qRT-PCR products were analyzed by melting curve analysis.

For the analysis of miRNA array data, a t-test was used to determine whether there were any significant miRNA expression differences between TOF patients and healthy controls. P-values < 0.05 were considered statistically significant. Significant data were further analyzed by clustering and expression profiles were visualized with GeneSpring 10.0 (Agilent Technology). For qRT-PCR analysis, the relative expression level for each miRNA or mRNA was calculated using the $2^{-\Delta\Delta Ct}$ method. To account for possible differences input amount, miRNA expression was normalized to

U6 while mRNAs expression was normalized to *GAPDH*.

Primer sequences for gene expression detection.

MEF2C (F-5-agatacccacaacacaccacgcgcc-3; R-5-atccttcagagagtcgcatgcgctt-3);

GATA-4 (F- 5-gacaatctggttaggggaagc-3; R-5-accagcagcagcgaggagat-3);

Nkx-2.5 (F-5-cgccgctccagttcatag-3; R-5-ggtggagctggagaagacaga-3);

TropT (F-5-gtgggaagaggcagactgag-3; R-5-atagatgctctgccacagc-3);

bMHC (F-5-gaagcccagcacatcaaaag-3; R-5-gatcaccaacaacccctacg-3);

cActin (F-5-tcctgatgcgcatttttattc-3; R-5-aacaccactgctctagccacg-3);

α-SMA (F-5-ccagctatgtgaagaagaagagg-3; R-5-gtgatctccttctgcattcggt-3);

calponin (F-5-ttttgaggccaacgacctgt-3; R-5-tcctttcgtcttcgccatg-3) ;

Flow Cytometry for apoptosis and necrosis detection. Apoptosis and necrosis were detected by staining for Annexin V and PI (Roche, Switzerland) as previously described [1]. In Brief, 10^6 cells were washed with PBS and centrifuged at 200 g for 5 min. After that, the cell pellet was re-suspended in 100 μl of Annexin-VFLUOS labeling solution and incubated at 25°C for 15 min. Then 0.5 ml incubation buffer was added and the mixture was analyzed on a MoFlo XDP flow cytometry (Beckman Coulter, Inc.) using a 488 nm excitation and a 515 nm filter for fluorescein detection and a 600 nm filter for PI detection.

miRNA-940 target gene prediction. GOmir was used to identify potential human

miRNA-940 target genes. GOmir integrates miRNA target prediction and functional analysis by combining the predicted target genes from TargetScan, miRanda, RNAhybrid and PicTar 4-way databases and provides a full genedescription and functional analysis for each target gene. Only those genes that were predicted by all of the tools and have confirmed roles in development, differentiation, proliferation, cell growth or cell cycle by previous studies were considered as candidates. We assumed that the consensus among databases that predicted any given gene as a target indicated the likelihood that the miRNA-mRNA interaction would be relevant [2].

Since TOF was a manifestation of abnormal heart development, we focus those predicted genes that have been shown to affect heart development. GOmir identified JARID2 as potential target genes for miRNA-940 and JARID2 has been shown to regulate outflow tract morphogenesis. So we focus on JARID2 for the downstream analysis. Two independent strategies were used to confirm these predicted genes as the targets of miRNA-940. First, a luciferase reporter assay was used to confirm the target gene. For construction of the luciferase reporter plasmid of JARID2, a 1103 bp fragment which contains predicted target binding site (according to http://pictar.mdc-berlin.de) in 3′ UTR of JARID2 was amplified from human genomic DNA by PCR using primers (JARID2 primer: F: 5'-ACTAGTCACTATGCATCTGTTCCAGG-3'; R: 5'-AAGCTTGTGCAGAGCAGCGCTAATAA-3') and inserted into the 3′UTR of a cytomegalovirus-driven firefly luciferase gene. The predicted binding site (CCTGCCT and CTGCCTA) in 3′ UTR of JARID2 was deleted as a contrast. HEK293 cells were

transfected with luciferase reporter plasmids and the miRNA-940 mimics (30nM), and a Renilla luciferase plasmid was cotransfected as an internal control. Cells were harvested 48 h after transfection. Luciferase activities were measured using dual luciferase reporter assay system (Promega). Relative luciferase expression was measured on a scintillation counter by using a dual luciferase reporter system. Second, to confirm that miRNA-940 could endogenously regulate JARID2 expression, hCMPCs were transfected with miRNA-940 mimics, miRNA-940 inhibitor or negative control. After 48h, the protein expression level of JARID2 was detected by western blot (R&D, USA). JARID2 protein levels were further examined in human tissues follow the methods mentioned above.

Reference

1. Liu J, van Mil A, Vrijsen K, et al. MicroRNA-155 prevents necrotic cell death in human cardiomyocyte progenitor cells via targeting RIP1. J Cell Mol Med 2011;15:1474-82.

2. Ventura A, Young AG, Winslow MM, et al. Targeted deletion reveals essential and overlapping functions of the miR-17 through 92 family of miRNA clusters. Cell 2008;132:875-86.

Supplemental Figure Legends

Supplemental Figure 1. Islet-1 staining of Sca-1$^+$ hCMPCs. Isolated Sca-1$^+$ hCMPCs was stained for Islet-1 (Isl-1), and DAPI. Merge shows the Sca-1$^+$ hCMPC expressed Islet-1, a marker of cardiac progenitor cells derived from the second heart field (×200).

Supplemental Figure 2. Efficiency of the miRNA-940 transfectrion. (**A**) FAM staining showed the transfected cells of a typical field under the microscope (×100). (**B**) Quantification of RT-PCR results examining the miRNA-940 expression after cells transfected with control, miRNA-940 mimics and inhibitor, n=6.

Supplemental Figure 3. miRNA-940 mimics and inhibitor do not affect human cardiomyocyte progenitor cells viability. Cell viability was assayed and quantified by measuring CCK-8 absorbance, n=7.

Supplemental Figure 4. miRNA-940 mimics and inhibitor do not cause apoptosis and necrosis. Annexin V (X-axis) and PI (Y-axis) staining for (**A**) Mock; (**B**) miRNA-940 mimics (30 nM); (**C**) miRNA-940 inhibitors treatments (30 nM) are shown. UL, upper left; UR, upper right; LL, lower left; LR, low right.

Supplemental Figure 5. miRNA-940 expression during hCMPC differentiation. Quantification of miRNA-940 expression during inducing hCMPCs differentiate to

cardiomyocytes at 8 different time points. RT-PCR was performed for 4 to 6 times at each time point.

Supplemental Figure 6. miRNA-940 mimics and inhibitor do not affect cardiac-specific gene expression under differentiation condition. Relative mRNA levels of (**A**) GATA4; (**B**) MEF2C; (**C**) Nkx2.5; (**D**) β-MHC; (**E**) cActin; (**F**) TropT as assessed by qRT-PCRs, n=5.

Supplemental Figure 7. Effects of miRNA-940 mimics and inhibitor on differentiation of hCMPCs to cardiomyocyte. (**A**) Representative α-actinin and NKX2.5 staining pictures of miRNA-940 mimics and inhibitor transfected human cardiomyocyte progenitor cell under differentiation condition (×200). (**B**) Quantification of miRNA-940 mimics and inhibitor on the degree of human cardiomyocyte progenitor cell differentiation.

Supplemental Figure 8. Effects of miRNA-940 mimics and inhibitors on differentiation of hCMPCs to smooth muscle cells. (**A**) Relative mRNA levels of α-SMA. (**B**) Relative mRNA levels of calponin. (**C**) Representative Western-Blot results for the effects of miRNA-940 on α-SMA and calponin protein expression. (D&E) Relative protein levels of α-SMA (**D**) and calponin (**E**). Similar results were observed in 5 independent experiments. *P < 0.05, compared to control.

Supplemental Figure 9. Effects of miRNA-940 mimics and inhibitor on hCMPCs migration as assayed by the chamber assay. (A) Representative images showing the effects of miRNA-940 on hCMPCs migration. (B) Quantification of the effects of the miRNA-940 on hCMPCs migration . *P < 0.05, compared to mock. mock, n=5; mimics, n=5; inhibitor, n=5.

Figure S1

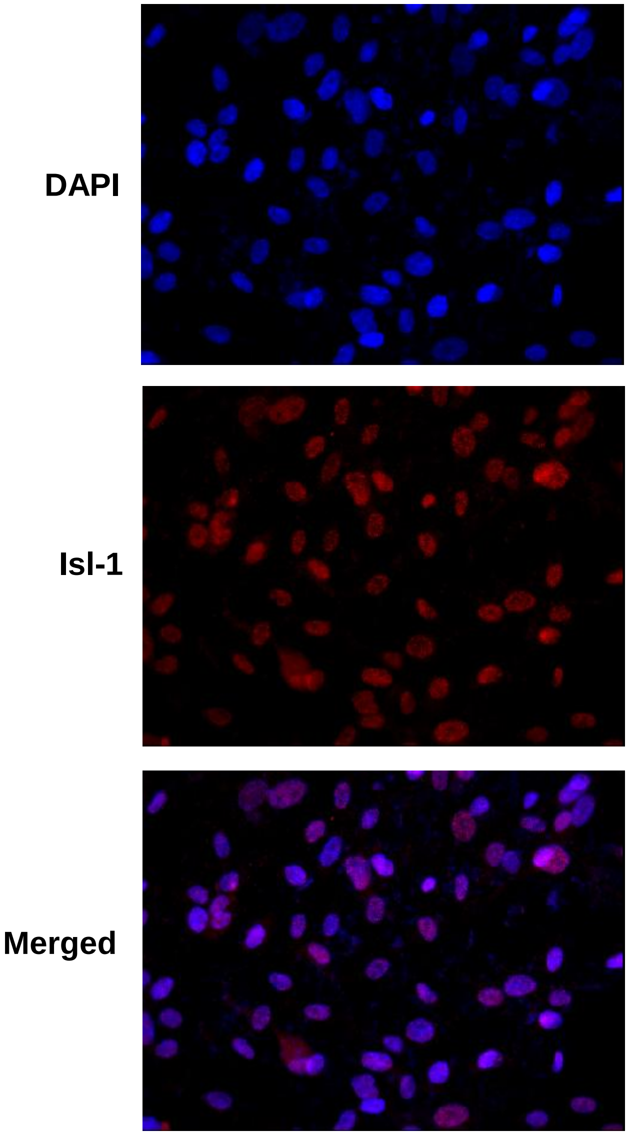

Figure S2

A

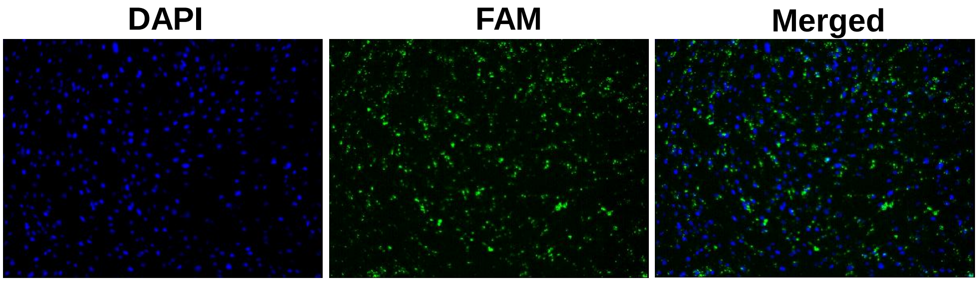
DAPI
FAM
Merged

B

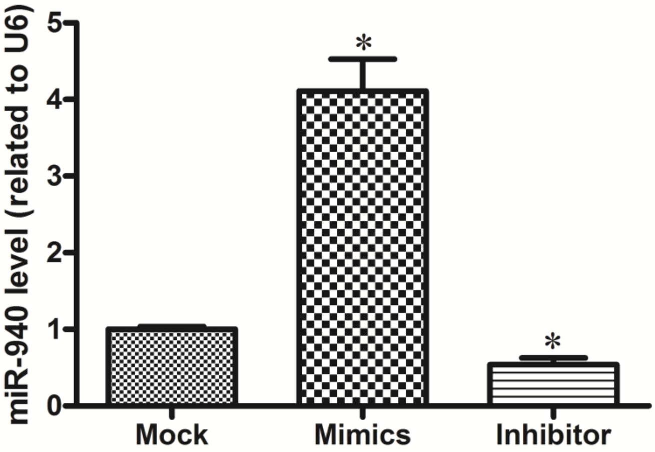
miR-940 level (related to U6)
0
1
2
3
4
5
*
*
Mock
Mimics
Inhibitor

Figure S3

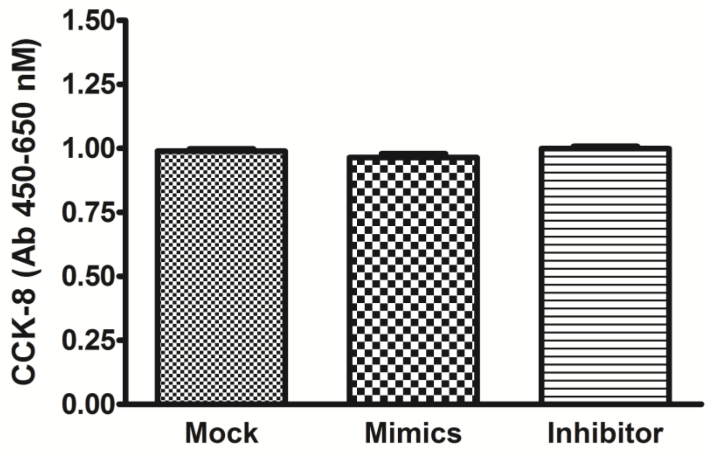

CCK-8 (Ab 450-650 nM)
1.50
1.25
1.00
0.75
0.50
0.25
0.00
Mock
Mimics
Inhibitor

Figure S4

A

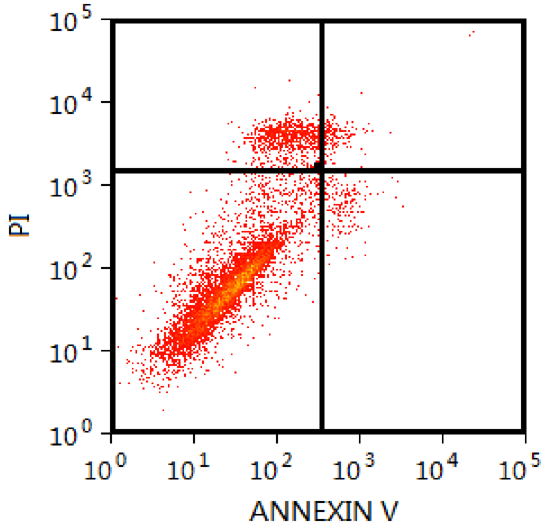

Quad	% Gated
UL	10.12
UR	1.83
LL	86.13
LR	1.92

B

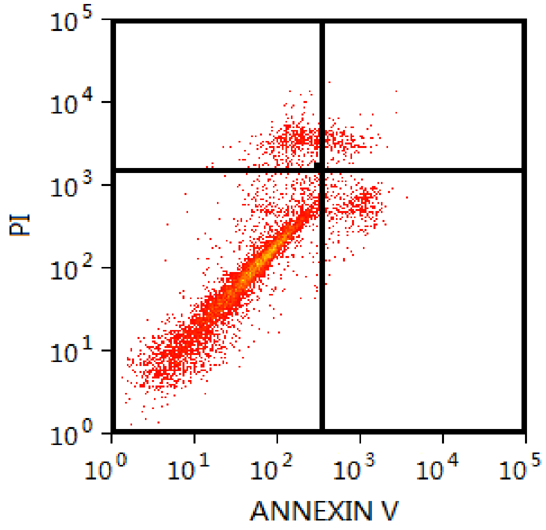

Quad	% Gated
UL	5.78
UR	2.30
LL	87.46
LR	4.46

C

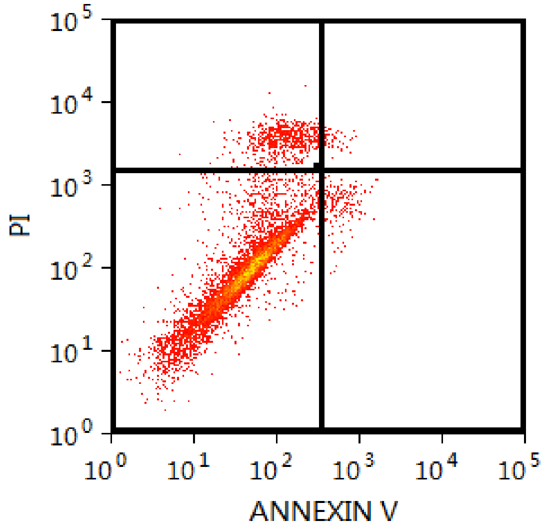

Quad	% Gated
UL	8.33
UR	0.56
LL	89.16
LR	1.95

Figure S5

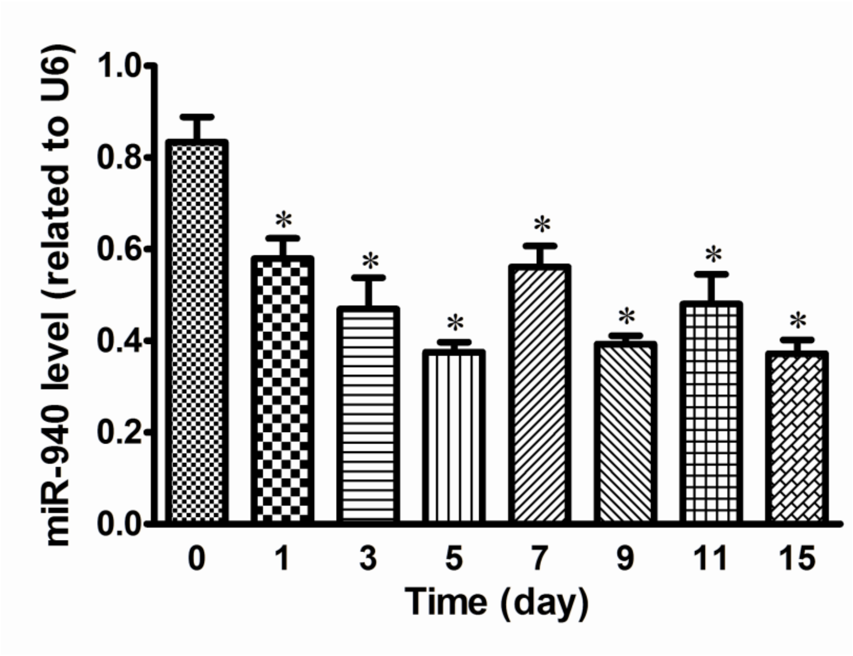

miR-940 level (related to U6)
1.0
0.8
0.6
0.4
0.2
0.0
0
1
3
5
7
9
11
15
Time (day)

Figure S6

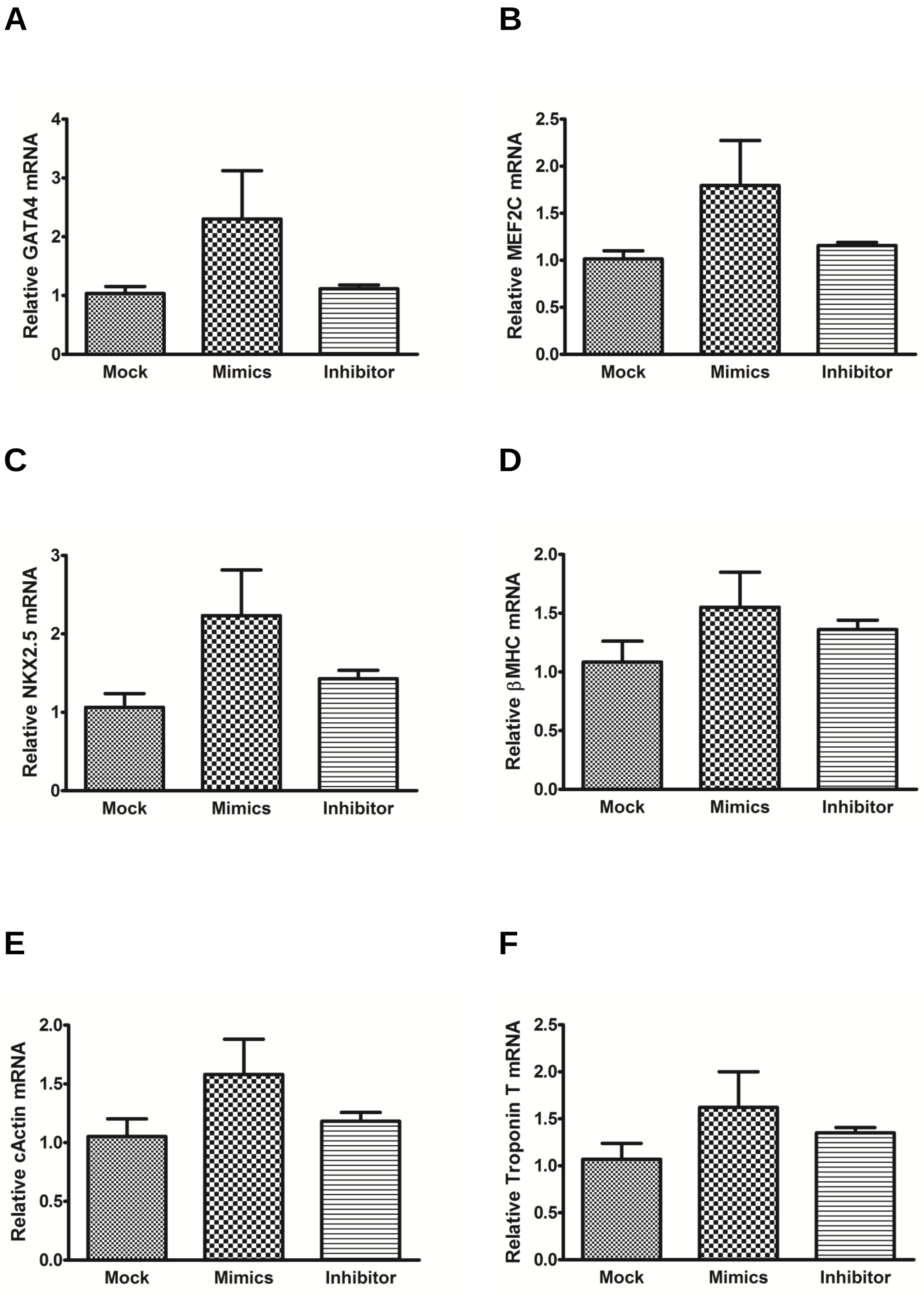
A
Relative GATA4 mRNA
0
1
2
3
4
Mock
Mimics
Inhibitor
B
Relative MEF2C mRNA
0.0
0.5
1.0
1.5
2.0
2.5
Mock
Mimics
Inhibitor
C
Relative NKX2.5 mRNA
0
1
2
3
Mock
Mimics
Inhibitor
D
Relative βMHC mRNA
0.0
0.5
1.0
1.5
2.0
Mock
Mimics
Inhibitor
E
Relative cActin mRNA
0.0
0.5
1.0
1.5
2.0
Mock
Mimics
Inhibitor
F
Relative Troponin T mRNA
0.0
0.5
1.0
1.5
2.0
2.5
Mock
Mimics
Inhibitor

Figure S7

A

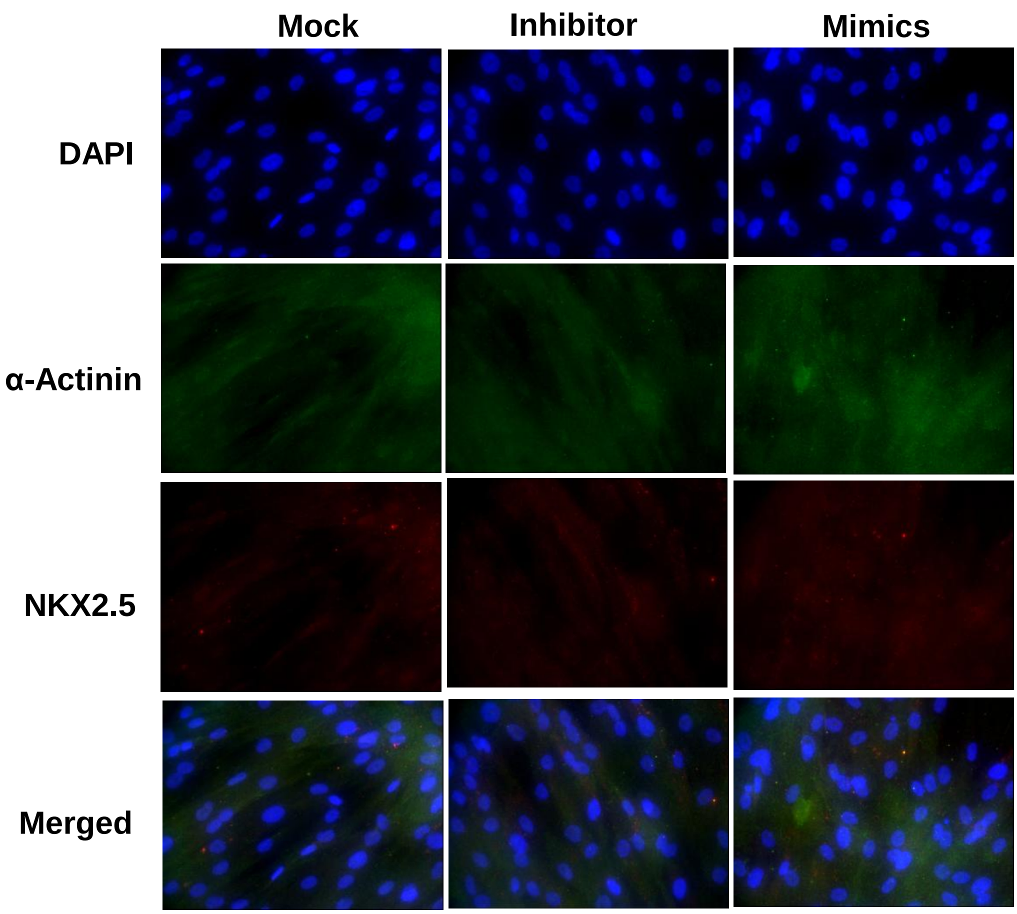

B

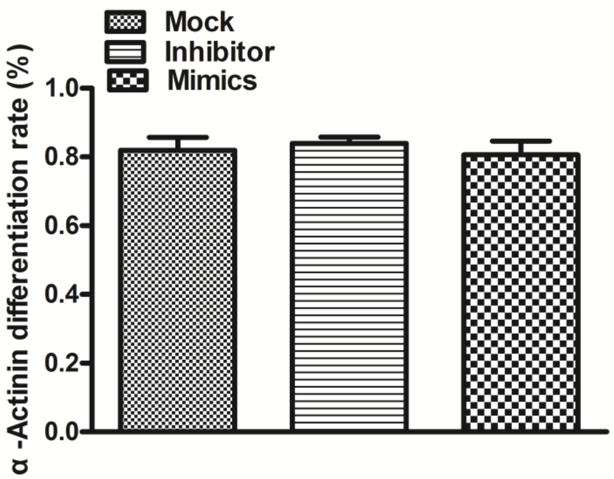

C

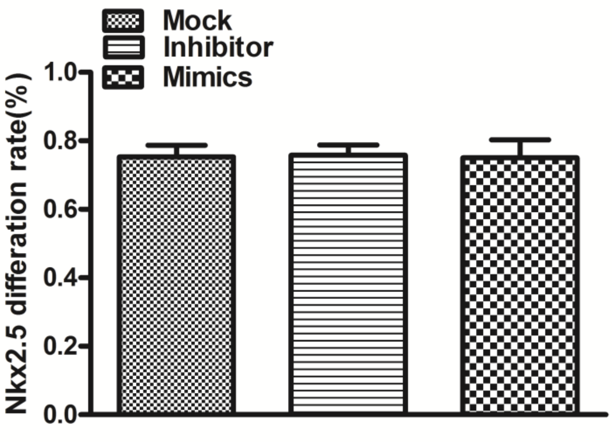

Figure S8

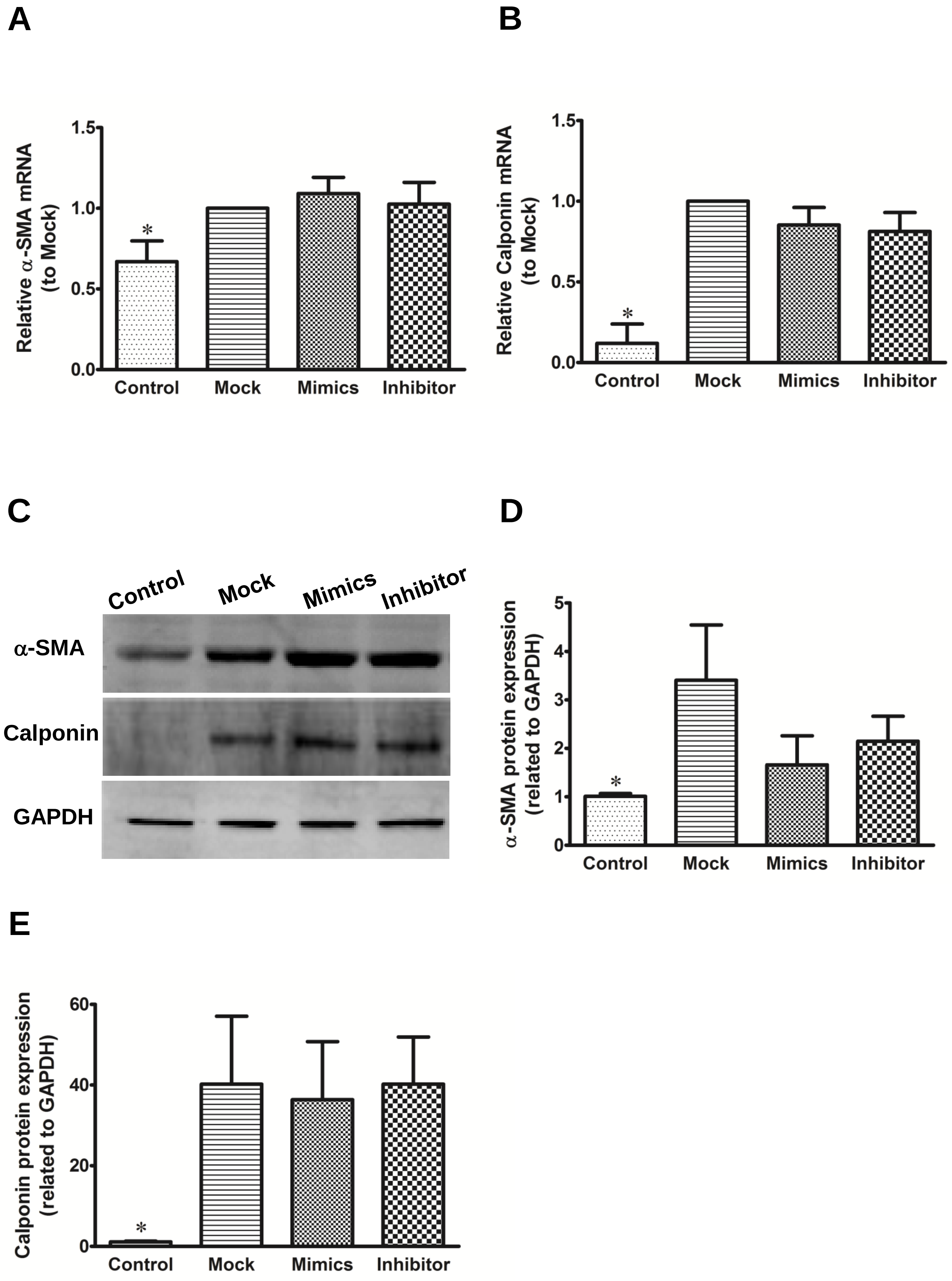

A
Relative α-SMA mRNA (to Mock)
1.5
1.0
0.5
0.0
*
Control
Mock
Mimics
Inhibitor
B
Relative Calponin mRNA (to Mock)
1.5
1.0
0.5
0.0
*
Control
Mock
Mimics
Inhibitor
C
Control
Mock
Mimics
Inhibitor
α-SMA
Calponin
GAPDH
D
α-SMA protein expression (related to GAPDH)
5
4
3
2
1
0
*
Control
Mock
Mimics
Inhibitor
E
Calponin protein expression (related to GAPDH)
60
40
20
0
*
Control
Mock
Mimics
Inhibitor

Figure S9

A

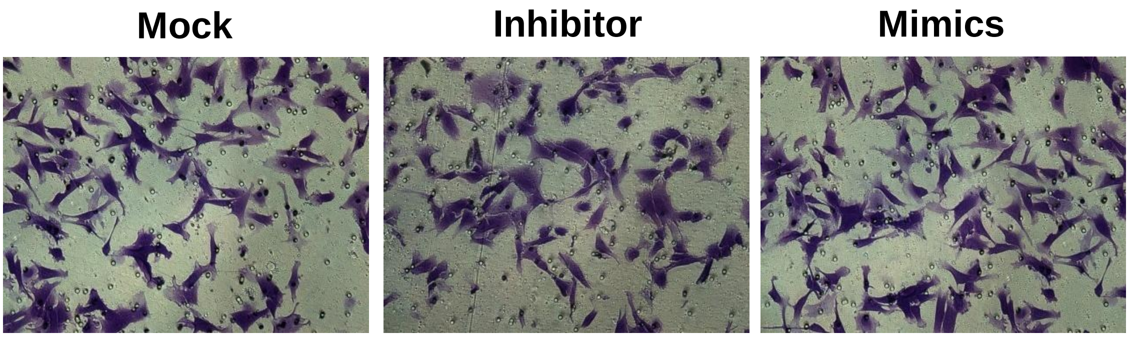
Mock
Inhibitor
Mimics

B

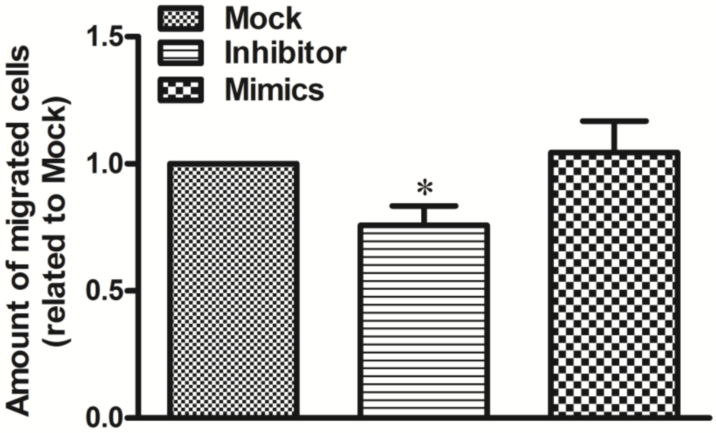
Mock
Inhibitor
Mimics
1.5
1.0
0.5
0.0
Amount of migrated cells
(related to Mock)
*

Supplementary Table 1 Differentially expressed miRNAs between Tetralogy of Fallot patients and healthy controls

Systematic Name	P value	Fold Change	Regulation
hsa-miR-940	0.000	4.028	Down
hsa-miR-10a	0.015	2.562	Down
hsa-miR-134	0.000	2.498	Down
hsa-miR-451	0.027	1.984	Down
hsa-miR-144*	0.037	1.751	Down
hsa-miR-136	0.000	1.733	Down
hsa-miR-148a	0.004	1.677	Down
hsa-miR-1281	0.001	1.618	Down
hsa-miR-886-3p	0.013	1.574	Down
hsa-miR-193a-3p	0.002	1.556	Down
hsa-miR-365	0.003	1.551	Down
hsa-miR-193a-5p	0.006	1.478	Down
hsa-miR-377	0.002	1.424	Down
hsa-miR-193b	0.001	1.419	Down
hsa-miR-1207-5p	0.015	1.399	Down
hsa-miR-720	0.001	1.375	Down
hsa-miR-127-3p	0.005	1.360	Down
hsa-miR-139-5p	0.014	1.351	Down
hsa-miR-376c	0.004	1.322	Down
hsa-miR-30c-1*	0.013	1.318	Down
hsa-miR-584	0.004	1.313	Down
hsa-miR-376a	0.006	1.307	Down
hsa-miR-299-5p	0.032	1.292	Down
hsa-miR-381	0.027	1.279	Down
hsa-miR-574-5p	0.004	1.274	Down
hsa-miR-22	0.041	1.264	Down

hsa-miR-654-3p	0.025	1.225	Down
hsa-miR-204	0.000	3.624	Up
hsa-miR-181a-2*	0.000	2.105	Up
hsa-miR-221*	0.050	2.059	Up
hsa-miR-222	0.006	2.047	Up
hsa-miR-155	0.000	2.018	Up
hsa-miR-513a-5p	0.012	2.003	Up
hsa-miR-146b-5p	0.003	1.903	Up
hsa-miR-130b	0.002	1.635	Up
hsa-miR-21	0.001	1.561	Up
hsa-miR-224	0.047	1.559	Up
hsa-miR-34b*	0.026	1.525	Up
hsa-miR-132	0.001	1.504	Up
hsa-miR-598	0.010	1.461	Up
hsa-miR-744	0.003	1.423	Up
hsa-miR-625	0.000	1.408	Up
hsa-miR-769-5p	0.002	1.383	Up
hsa-miR-887	0.039	1.362	Up
hsa-miR-497	0.000	1.357	Up
hsa-miR-181c	0.003	1.352	Up
hsa-miR-23b	0.001	1.349	Up
hsa-miR-28-5p	0.004	1.304	Up
hsa-miR-320c	0.000	1.303	Up
hsa-miR-129-3p	0.010	1.298	Up
hsa-miR-34a	0.034	1.289	Up
hsa-miR-652	0.034	1.285	Up
hsa-miR-27b	0.001	1.281	Up
hsa-miR-24	0.001	1.275	Up
hsa-miR-185	0.002	1.248	Up

hsa-miR-320b	0.003	1.245	Up
hsa-miR-148b	0.001	1.243	Up
hsa-miR-194	0.031	1.238	Up
hsa-miR-324-5p	0.002	1.236	Up
hsa-miR-93	0.006	1.229	Up
hsa-miR-320d	0.023	1.224	Up
hsa-miR-195	0.019	1.191	Up
hsa-miR-130a	0.005	1.191	Up
hsa-miR-320a	0.000	1.190	Up
hsa-miR-125b	0.008	1.184	Up
hsa-miR-505	0.004	1.177	Up
hsa-miR-125b-2*	0.005	1.162	Up
hsa-miR-331-3p	0.032	1.159	Up
hsa-miR-423-5p	0.008	1.156	Up
hsa-miR-99b	0.048	1.151	Up
hsa-miR-374b	0.011	1.148	Up
hsa-miR-361-5p	0.023	1.144	Up
hsa-miR-140-3p	0.018	1.128	Up
hsa-miR-186	0.009	1.112	Up
hsa-miR-151-3p	0.042	1.106	Up

Supplementary Table 2 microRNAs differentially expressed in the heart, pancreas and lung

Systematic Name	P value	Heart (Mean±SD)	Pancreas (Mean±SD)	Lung (Mean±SD)
hsa-miR-1	0.000	22137±2578	34±31	54±3
hsa-miR-940	0.000	11252±782	497±74	156±16
hsa-let-7a	0.000	7087±256	13221±1387	12536±350
hsa-let-7f	0.006	5566±519	7376±575	7749±635
hsa-miR-625*	0.000	5424±585	157±38	68±20
hsa-miR-26a	0.000	4412±405	6544±441	7617±258
hsa-let-7c	0.000	3804±142	10223±190	8358±421
hsa-miR-4274	0.000	3720±385	119±30	126±7
hsa-miR-3172	0.000	3653±686	932±119	165±7
hsa-miR-126	0.000	2976±161	628±53	5030±200
hsa-miR-23b	0.000	2724±236	1829±174	1531±98
hsa-miR-1238	0.000	2502±465	94±50	95±15
hsa-let-7d	0.000	2438±228	5523±118	4850±346
hsa-miR-484	0.000	2354±212	152±64	188±20
hsa-miR-1825	0.000	2210±780	201±41	147±11
hsa-miR-4281	0.000	1714±55	704±123	1937±45
hsa-miR-23a	0.001	1608±116	908±112	1415±141
hsa-miR-133a	0.000	1418±162	57±24	48±17
hsa-miR-1237	0.000	1395±377	77±42	100±5
hsa-miR-133b	0.000	1254±93	46±17	38±7
hsa-miR-1913	0.001	1069±491	168±53	137±18
hsa-miR-499-5p	0.001	915±73	16±6	25±21
hsa-let-7e	0.001	831±96	1161±62	1331±53
hsa-miR-638	0.000	829±85	3208±158	2427±208
hsa-miR-27b	0.000	824±102	886±74	380±23

hsa-miR-143	0.003	756±53	551±81	912±79
hsa-miR-4284	0.000	690±163	103±27	77±10
hsa-miR-1281	0.002	681±285	300±13	183±10
hsa-miR-1249	0.000	552±58	87±36	122±12
hsa-miR-1826	0.000	532±51	4895±444	990±50
hsa-miR-1979	0.000	458±26	7066±596	1050±50
hsa-let-7g	0.000	359±28	984±38	909±87
hsa-miR-145	0.001	322±25	398±116	880±52
hsa-miR-486-5p	0.004	267±25	123±58	491±35
hsa-let-7i	0.000	261±22	609±35	907±93
hsa-miR-30d	0.000	260±7	413±37	784±42
hsa-miR-16	0.000	248±14	879±71	3265±34
hsa-miR-195	0.000	242±22	506±67	882±22
hsa-miR-26b	0.000	225±10	734±61	1129±25
hsa-miR-3196	0.000	17724	2191±261	2046±113
hsa-miR-451	0.000	171±17	419±61	10284±311
hsa-miR-1975	0.000	166±6	1594±60	936±46
hsa-miR-2861	0.000	138±9	976±176	721±42
hsa-miR-92a	0.000	137±23	1047±54	968±32
hsa-miR-191	0.000	134±7	258±45	574±88
hsa-miR-15b	0.000	128±13	243±49	943±50
hsa-miR-21	0.000	122±11	355±66	2717±309
hsa-miR-762	0.000	97±9	216±36	535±33
hsa-miR-1915	0.000	93±1	875±161	567±45
hsa-miR-1308	0.000	53±5	473±108	186±2
hsa-miR-217	0.000	51±5	500±16	21±3
hsa-miR-1246	0.000	48±23	67±36	1665±124
hsa-miR-375	0.000	37±13	12461±527	102±2
hsa-miR-148a	0.000	28±6	3116±495	53±4

hsa-miR-200c	0.000	8 ±7	3903 ±743	1611 ±11
